# Olefin metathesis in nano-sized systems

**DOI:** 10.3762/bjoc.7.13

**Published:** 2011-01-19

**Authors:** Didier Astruc, Abdou K Diallo, Sylvain Gatard, Liyuan Liang, Cátia Ornelas, Victor Martinez, Denise Méry, Jaime Ruiz

**Affiliations:** 1 Institut des Sciences Moléculaires, UMR CNRS No 5255, Université Bordeaux 1, 351 Cours de la Libération, 33405 Talence Cedex, France

**Keywords:** dendrimer, green chemistry, metathesis, nano-system, water

## Abstract

The interplay between olefin metathesis and dendrimers and other nano systems is addressed in this mini review mostly based on the authors’ own contributions over the last decade. Two subjects are presented and discussed: (i) The catalysis of olefin metathesis by dendritic nano-catalysts via either covalent attachment (ROMP) or, more usefully, dendrimer encapsulation – ring closing metathesis (RCM), cross metathesis (CM), enyne metathesis reactions (EYM) – for reactions in water without a co-solvent and (ii) construction and functionalization of dendrimers by CM reactions.

## Introduction

Olefin metathesis reactions [1–7] have been successfully catalyzed under standard conditions, including reactions at room temperature and sometimes even in air, with commercial Grubbs-type catalysts [8–9]. These are now largely developed for industry with functional substrates for the synthesis of highly sophisticated pharmaceutical products and polymers. There is continuing research in the olefin metathesis field, however, because of the economical and ecological constraints of modern society. This requires that the catalyst amounts be as low as possible and that polluting classic organic solvents be replaced by “greener” solvents such as water or super-critical carbon dioxide. Therefore during the last decade, we have attempted to make progress in this field with dendrimers using nano-organometallic chemistry [10]. There are several ways in which dendrimer chemistry can be useful in this direction, and this short review article will indicate the various connections between metathesis reactions and dendrimer chemistry.

## Review

### Covalent attachment of the olefin metathesis catalyst to the tethers of the dendrimer periphery

**Scheme 1:**
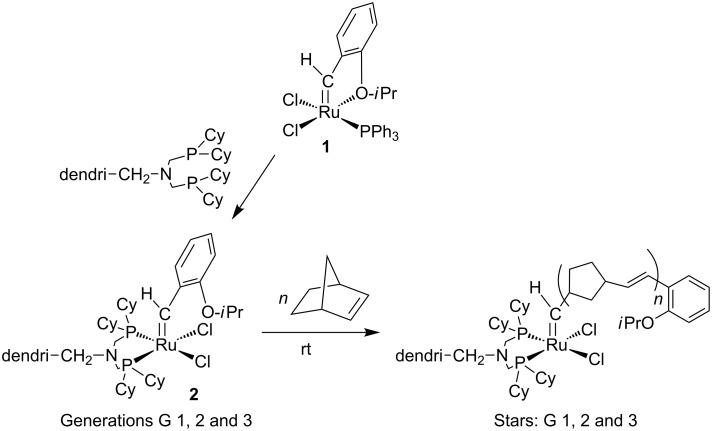
Strategy for the ROMP of norbornene by Ru-benzylidene dendrimers to form dendrimer-cored stars.

The attachment of catalysts to dendrimers was mostly focused on the recovery of the catalyst. Only a few metallodendritic carbene complexes with covalent binding of the olefin metathesis catalyst are known. Prior to our involvement only compounds with four branches were known [11–14] but good recyclability still remained a challenge. The difficulty resided in the need to sustain both metathesis activity and stability of the metallodendrimer. Thus, we selected the ruthenium family of catalysts and designed metallodendrimers containing ruthenium-benzylidene fragments located at the dendrimer periphery and chelating phosphine ligands on the branch termini. The choice of chelating phosphines may seem counter-intuitive, because the activity of Grubbs catalysts involves the decoordination of a phosphine from these *trans*-bis-phosphine complexes [15]. However, studies by the groups of Hofmann [16–18], Fog [19–20] and Leitner [20] had demonstrated the metathesis activity of *cis*-bis-phosphine ruthenium benzylidene catalysts. We therefore used Reetz’s bis-phosphines derived from the commercial polyamine DSM dendrimers [21]. These dendritic bis-phosphines are useful and versatile in metallodendritic catalysis and provided the first recyclable metallodendritic catalysts [21]. Moreover, dendritic bis-phosphines with two phenyl groups on each phosphorus atom very cleanly yielded the first dendrimers decorated with clusters at the periphery via an efficient electron-transfer-chain reaction using [Ru_3_(CO)_12_] catalyzed by [Fe^I^Cp(η^6^-C_6_Me_6_)] leading to the substitution of a carbonyl of the [Ru_3_(CO)_12_] by a dendritic phosphine on each tether [22]. Related dendritic bis-phosphines with two cyclohexyl groups on each phosphorus were decorated with ruthenium benzylidene metathesis functions using Hoveyda’s ruthenium benzylidene metathesis catalyst, **1** [23], as the starting point. These reactions provided four generations of new, stable metallodendrimers **2** containing ruthenium-benzylidene fragments at the periphery (Scheme 1) [24–25]. The fourth-generation metallodendrimer containing 32 ruthenium-benzylidene fragments, however, was found to have a rather low solubility in common organic solvents, unlike the three first-generation complexes that contained 4, 8 and 16 ruthenium-benzylidene moieties, respectively. The weak solubility of the 32-Ru dendrimer is presumably due to steric congestion at its periphery. Such steric congestion is also responsible for the decrease of the catalytic activity of Ru and Pd high-generation dendritic catalysts, even when these metallodendritic catalysts are soluble. The X-ray crystal structure of the model mononuclear complex in which the dendritic branch was replaced by a benzyl group showed distorted square pyramidal geometry and the classic geometric features of a Ru=C double bond. The oxygen atom of the isopropyl aryl ether group is not coordinated unlike in Hoveyda’s complex **1**. The fundamental organometallic chemistry of this monomeric model complex was also original [24–25].

**Scheme 2:**
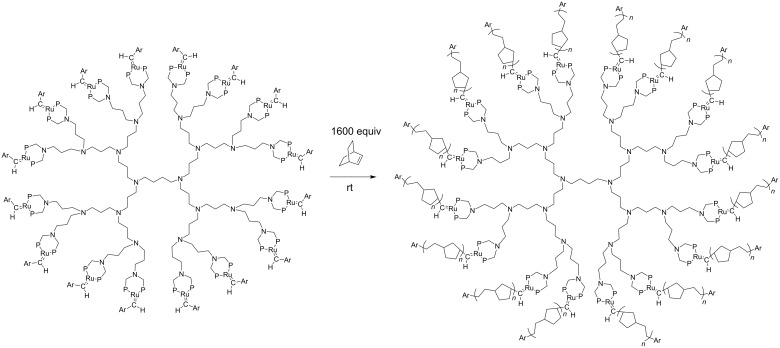
Third-generation (16 Ru atoms) ruthenium-benzylidene dendrimer that catalyzes the ROMP of norbornene at 25 °C to form dendrimer-cored stars.

The three first generations of metallodendrimers **2** and the model complex do not catalyze RCM reactions, but they were efficient catalysts for the ROMP of norbornene under ambient conditions, giving dendrimer-cored stars (Scheme 1 and Scheme 2) [24–25]. Analysis of the molecular weights by size exclusion chromatography gave data that were close to the theoretical values, which indicated that all the branches were efficiently polymerized. Dendritic-cored stars with an average of about 100 norbornene units on each dendritic branch were synthesized from the three first generations of ruthenium-carbene dendrimers containing 4, 8 and 16 Ru=C bonds, respectively.

Two kinds of dendritic effect were found on analysis of the kinetic data. First, the dendrimers were more efficient catalysts than the monomeric model complex. This could possibly be due to labilization of metal-phosphine bonds that is facilitated in dendrimers as compared to the monomer for entropic reasons. Indeed, DFT calculation showed that the catalytic process must involve decoordination of a phosphorus atom, since the interaction of the olefin with the diphosphine complex is non-bonding. The dendritic ruthenium-benzylidene dendrimers were air-sensitive in contrast to the monomer model complex, consistent with more rapid dissociation of the alkyl phosphine in the dendrimers than in the monomer. Secondly, the efficiency of catalysis decreased upon increasing the dendrimer generation. This second dendritic effect is thus a negative one, and it is probably related to the more difficult access to the metal center due to the increasing steric effect at the dendrimer periphery when the generation increases.

Analogous ruthenium benzylidene dendrimers were very recently synthesized with two *tert*-butyl groups on each phosphorus atom, and these were slightly more reactive ROMP catalysts for the polymerization of norbornene than those carrying cyclohexyl substituents [25]. These new dendritic ligands, in particular those of low generation (with up to 8 branches), also proved very efficient in palladium catalysis [26–31].

### Construction and decoration of dendrimers using olefin metathesis reactions

**Scheme 3:**
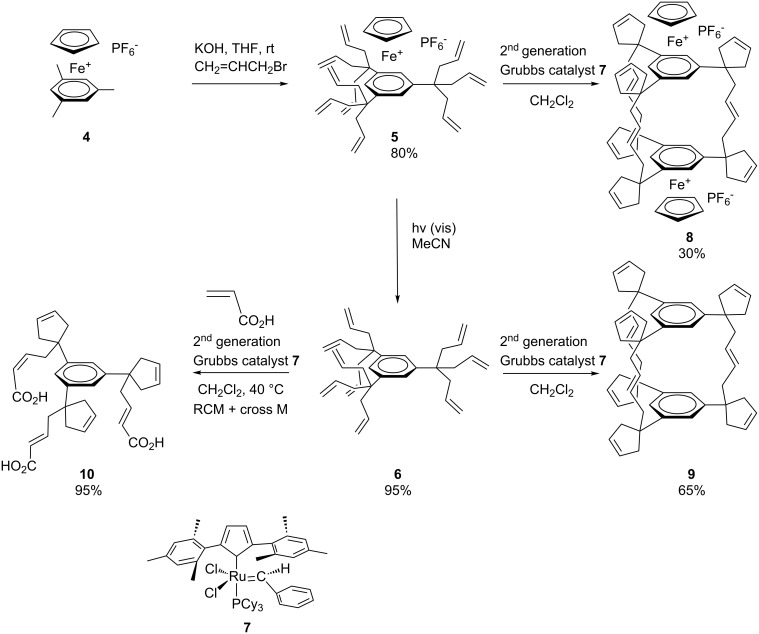
Multiple carbon–carbon bond formation upon RCM and CM, and the complete switch of selectivity in the presence of acrylic acid.

Star-shaped and dendrimer compounds that are terminated by carbon–carbon double bonds can undergo CM reactions with olefins. To begin with, we examined cross olefin metathesis reactions with rather small aromatic molecules bearing a few double bonds, then continued the study with larger analogues. Temporary coordination of arenes to the strongly electron-withdrawing cationic 12-electron group CpFe^+^ greatly increases the acidity of its benzylic protons (the p*K*_a_ values of the arenes in DMSO are lowered upon complexation with CpFe^+^ by approximately 15 units, for instance from 43 to 28 in the case of C_6_Me_6_) [32–33]. Therefore, deprotonation of the CpFe(arene)^+^ complexes is feasible under mild conditions with KOH. Deprotonated CpFe(arene)^+^ complexes are good nucleophiles, and reactions with electrophiles such as the alkyl halides lead to the formation of new C–C bonds. Coupling the deprotonation and the nucleophilic reactions in situ in the presence of excess substrates leads to perfunctionalization in cascade multi-step reactions [34–35]. When the electrophile is allyl bromide, polyolefin compounds are produced after decomplexation by visible-light photolysis which removes the temporary activating CpFe^+^ group [36–38]. These compounds are then ideal substrates for RCM and CM. New structures were obtained using this strategy with durene, *p*-xylene, mesitylene, and pentamethylcobalticinium [39–41]. The latter was perallylated to yield a deca-allylated cobalticinium, and then RCM of the organometallic complex proceeded to afford a pentacyclopentylcyclopentadienyl Co sandwich complex using the first-generation Grubbs catalyst [Ru(PCy_3_)_2_Cl_2_(=CHPh)], **3**. Activation of mesitylene by the CpFe^+^ moiety in **4**, followed by a one-pot perallylation yielded [CpFe(nonaallylmesitylene)^+^][PF_6_^-^], **5,** from which the free arene derivative **6** was obtained on visible-light photolytic decomplexation [34–35,42]. First, a triple RCM reaction catalyzed by **3** proceeded in ten minutes under ambient condition, to afford an intermediate tetracyclic iron arene complex. Furthermore and interestingly, when the metathesis reaction was carried out in refluxing dichloroethane with the addition of the second-generation Grubbs catalyst [RuCl_2_(=CHPh)(bis-*N*-mesityl-NHC)], **7**, (Scheme 3, NHC = N-heterocyclic carbene), the di-iron cage compound **8** was formed. Similarly, the iron-free nonaallylated compound **6** gave, by metathesis catalyzed by **7**, the organic cage **9**. After hydrogenation with H_2_/Pd/C in CH_2_Cl_2_ of the tripled-bridged cage **9**, a single hydrogenated product was isolated. Another very useful feature is that the organic cage formation can be totally inhibited in the presence of acrylic acid to produce the triacid **10** by a more rapid stereoselective CM (Scheme 3) [43–44].

**Scheme 4:**
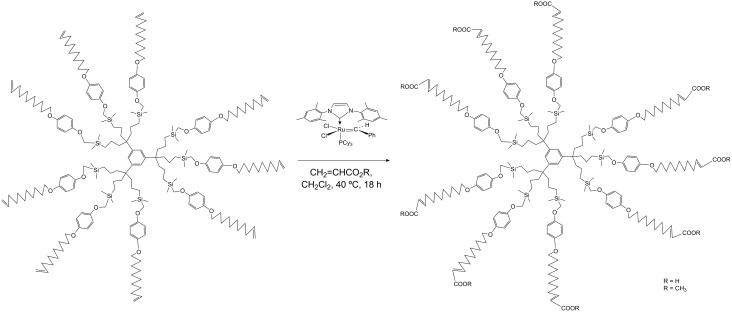
Example of chemo-, regio- and stereoselective CM of polyolefin dendrimers catalyzed by the 2^nd^ generation Grubbs metathesis catalyst to produce water-soluble dendrimers (R = H or CH_3_).

**Scheme 5:**
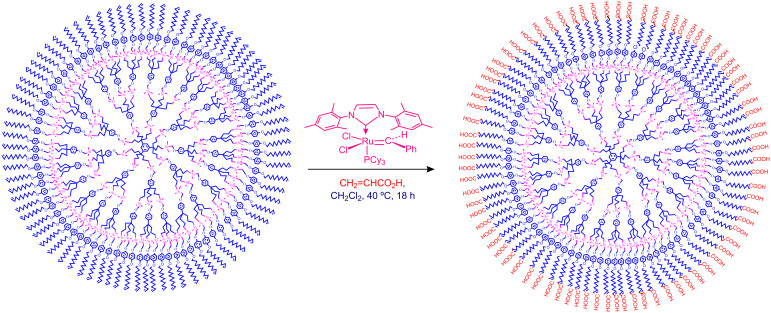
Example of chemo-, regio- and stereoselective CM of polyolefin dendrimers catalyzed by the 2^nd^ generation Grubbs metathesis catalyst: 81-tethered dendrimers.

Since successful CM with acrylic acid gave water-soluble compounds, this reaction was exploited to synthesize water-soluble dendrimers with carboxylate termini. Dendritic precursors were prepared with long tethers containing olefin termini so that no competitive RCM occurred unlike in the preceding example. Indeed, CM of these long-chain polyolefin dendrimers catalyzed by the 2^nd^ generation Grubbs metathesis catalyst **7** proceeded selectively to produce dendrimers whose tethers were terminated by carboxylic acid groups (Scheme 4 and Scheme 5). The corresponding carboxylates are water-soluble. Higher-generation dendrimers with carboxylic acid termini have been synthesized similarly [43–44].

Other attempts have been reported in the literature for the metathesis of polyolefin dendrimers or star compounds from which ring-closing metathesis products were formed. For instance, a third generation Fréchet-type dendrimer containing 24 allyl ether end groups was synthesized by the Zimmerman group, cross-linked using the RCM reaction, and the core removed hydrolytically without any significant fragmentation [45–47]. The results are analogous to those previously reported for homoallyl ether dendrimers suggesting that the less readily available homoallyl ether dendrimers can be replaced by their allyl ether analogues. The strategy consisting of performing RCM of branches and then to remove the core has also been applied by the Peng group to produce nanoparticle-cored dendrimers [48–51].

**Scheme 6:**
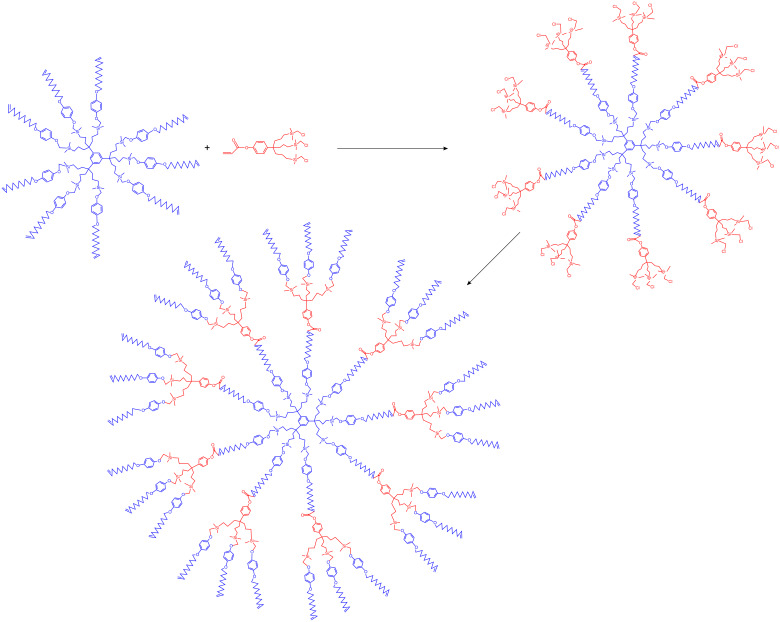
Dendrimer construction scheme from a 9-olefinic dendrimer to a 27-olefinic dendrimer by regio-and stereoselective CM using Grubbs second generation catalyst in CH_2_Cl_2_ at 40 °C, followed by a Wiliamson reaction with *p*-HOC_6_H_4_-O(CH_2_)_8_CH=CH_2_ in DMF at 80 °C. The next iteration of identical reaction sequence yields the 81-olefinic dendrimer.

Dendrimers have been synthesized by reaction sequences involving hydrosilylation of olefin-terminated dendrimer cores followed by Williamson reactions with the phenol triallyl dendron *p*-HOC_6_H_4_C(CH_2_CH=CH_2_)_3_ and iterations [42,52–53]. This allowed the building of large dendrimers and the extension of their tethers with alkenyl termini. CM of these large olefin-terminated dendrimers with acrylic acid was carried out in order to synthesize dendrimers terminated by carboxy groups (Scheme 5). These CM reactions were also extended to acrylates that contained a dendronic group. This strategy allowed constructing dendrimers from one generation to the next. Thus, iteration allows synthesizing a dendrimer of second generation with 81 olefin termini from a dendritic core containing 9 allyl termini after two iterative metathesis-hydrosilylation reactions (Scheme 6). This principle has also been extended to polymers and gold nanoparticles [54].

### Dendrimer-induced olefin metathesis in water

**Scheme 7:**
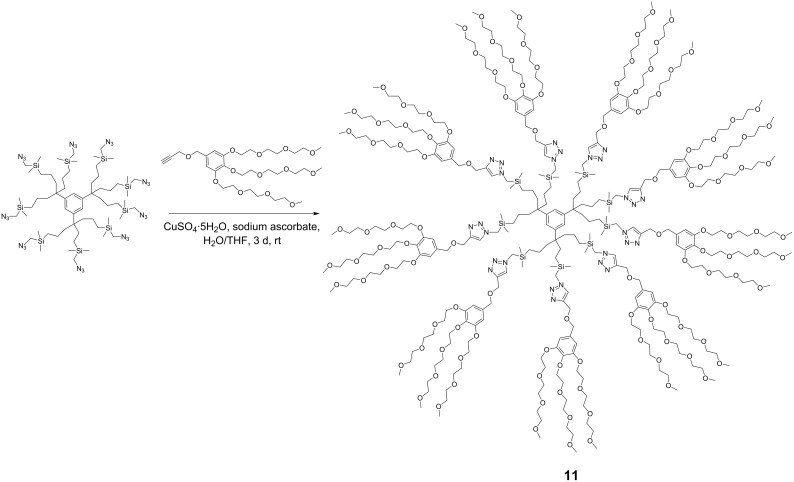
Synthesis of the water-soluble dendritic nanoreactor **7** for olefin metathesis in water without co-solvent.

Olefin metathesis of hydrophobic substrates, which are the large majority, in water instead of organic solvents is an obvious challenge that has been actively pursued [54–57] with water-soluble ruthenium catalysts [54], surfactants [58] and sonochemistry [59–62]. Using a low amount (0.083 mol %) of dendrimer, we have induced efficient olefin metathesis catalysis in water and with down to 0.04 % of the second-generation Grubbs catalyst **7** for RCM, (Table 1) [63]. The dendrimer **11** contains triethylene glycol termini that solubilize it in water. In this way, the dendrimer serves as a molecular micelle [64–65] to solubilize the hydrophobic catalysts and substrate in the hydrophobic interior of the nanoreactor. Its “click” synthesis is shown in Scheme 7.

**Table 1:** Compared RCM and EYM catalyzed by **7** in water without co-solvent, in the presence and absence of dendrimer **11**.

	Substrate	Product	Mol % Cat. **7**^a^	Conv. (%) with 0% den. **11**	Conv. (%) with 0.083% den. **11**

A	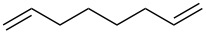	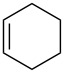	0.1	0	86^b^
B	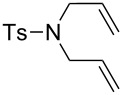	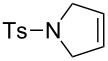	0.1 0.06 0.04	0 0 0	90^c^ 66^c^ 62^c^
C	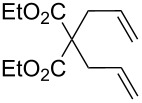	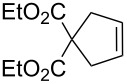	0.1	6^b^	89^b^
D	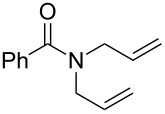	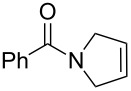	0.1	0	90^c^
E	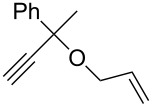	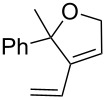	2	27^c^	97^c^
F	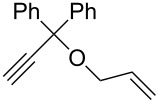	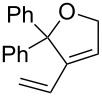	2	30^c^	99^c^

^a^The mol % catalyst **7** are pseudo-concentrations (rather than actual concentrations because **2** is insoluble in water; for instance, 4 mg of **7** dispersed in 47 mg of water, which corresponds to 0.1 mol % **7**). The dendrimer amount of 0.083 mol % corresponds to 28 mg. ^b^The reaction mixture without the catalyst was analyzed by ^1^H NMR in CDCl_3_, following filtration of the Ru catalyst or resulting residual species and subsequent extraction with ether. ^c^The reaction mixture without the catalyst was analyzed by GC (injection of the ether extract).

CM and EYM are also much favored by the presence of 0.083% mol of the dendrimer **11**, although these reactions still need 2% of Grubbs catalyst **7** [63], which is much more than the amount used for RCM.

RCM reactions can proceed in the presence of water even without surfactant, but the amount of 1^st^- or 2^nd^-generation Grubbs catalyst required then reaches 4 to 5% for good to high-yield reactions [66–67], which is of the order of 100 times more ruthenium catalyst than under our reaction conditions [63]. We have verified that these literature results [64–65] are reproducible with **7**.

Another key feature of the system is that the aqueous solution of the water-soluble dendrimer **11** can be recycled because **11** is insoluble in ether. Re-use of the aqueous solution of **11** is possible after subsequent filtration of the water-insoluble catalyst **7** after the reaction and removal of the organic reaction product by decantation or by extraction with ether. We have been able to recycle this aqueous dendrimer solution at least ten times without any significant decrease in yield. We have tested the stability of the Grubbs-II catalyst **7** in the presence of water at ambient temperature for 24 h, and found that it is stable in the absence of an olefin substrate. For example, after stirring a suspension of **7 (**0.1 mol %) in water for one day at 25 °C in air, the substrate and the dendrimer **11** were added and, after an additional day, the results of the RCM reaction were not significantly changed (80% conversion) compared to the result indicated in Table 1, entry B (90% conversion) under the same conditions. This means that the pre-catalyst **7** itself is stable and that the relative instability of **7** during metathesis in the presence of water (but in the absence of dendrimer **11**) is due to the slow decomposition of the catalytically active species formed during the RCM catalytic cycle. In particular, it has been shown that the methylene species [Ru(=CH_2_)Cl_2_{1,3-bis(mesityl)-NHC}(PCy_3_)], generated in the catalytic cycle of RCM reactions involving terminal olefins, is usually highly susceptible to dimerization and decomposition in CH_2_Cl_2_ or C_6_H_6_ [1]. Whatever the decomposition path of this species in the presence of water might be, it appears that the decomposition is considerably reduced when the dendrimer **11** is used for the RCM reactions. This strongly argues in favor of dendritic protection (probably by encapsulation) of the reactive species. RCM reactions need less catalyst **7** in organic solvents [1] than in the presence of water, especially in the absence of the dendrimer **11**. Thus the hydrophobic dendrimer interior should indeed favor the protection this intermediate ruthenium-methylene species from side reactions occurring in the presence of water.

## Conclusion

Olefin metathesis reactions are powerful methods that can be used for the construction of dendrimers and their functionalization with water-solubilizing carboxylate groups and other termini. In turn, water-soluble dendrimers can be used as molecular micelles as exemplified here. The implication of dendrimers in olefin metathesis reactions has mainly been focused on recovering the catalyst by loading the dendrimer with a functionalized catalyst. This strategy has been of very little success, because the % of catalyst used in metathesis reactions was rather high. This is due to the reactivity of methylene-metal intermediates that leads to side reactions. Consequently, another strategy involves protecting the catalytic intermediate in nanoreactors. Dendrimers are shown here to be excellent reactors achieving the goal of decreasing the catalytic amount when water is used as solvent. The success of using water as a reaction medium, even without co-solvent, is important in avoiding polluting organic solvents. Moreover, a very low Ru catalyst loading is possible in RCM with the fully recyclable water solution of the dendritic nanoreactor.
